# Angiopoietin-like protein 2 inhibits thrombus formation

**DOI:** 10.1007/s11010-024-05034-9

**Published:** 2024-06-16

**Authors:** Tiantian Zhang, Mingliang Zhang, Lingyu Guo, Dongsheng Liu, Kandi Zhang, Changlong Bi, Peng Zhang, Jin Wang, Yuqi Fan, Qing He, Alex C. Y. Chang, Junfeng Zhang

**Affiliations:** 1 Department of Cardiology, Shanghai Ninth People’s Hospital, Shanghai JiaoTong University School of Medicine, Shanghai, China; 2 Shanghai Institute of Precision Medicine, Shanghai Ninth People’s Hospital, Shanghai JiaoTong University School of Medicine, Shanghai, China

**Keywords:** Angiopoietin-like protein 2, Thrombosis, Platelet, ITIM (immunoreceptor tyrosine-based inhibition motif), ITAM (immunoreceptor tyrosine-based activation motif)

## Abstract

Acute myocardial infarction is mainly caused by a lack of blood flood in the coronary artery. Angiopoietin-like protein 2 (ANGPTL2) induces platelet activation and thrombus formation in vitro through binding with immunoglobulin-like receptor B, an immunoglobulin superfamily receptor. However, the mechanism by which it regulates platelet function in vivo remains unclear. In this study, we investigated the role of ANGPTL2 during thrombosis in relationship with ST-segment elevation myocardial infarction (STEMI) with spontaneous recanalization (SR). In a cohort of 276 male and female patients, we measured plasma ANGPTL2 protein levels. Using male *Angptl2*-knockout and wild-type mice, we examined the inhibitory effect of Angptl2 on thrombosis and platelet activation both in vivo and ex vivo. We found that plasma and platelet ANGPTL2 levels were elevated in patients with STEMI with SR compared to those in non-SR (NSR) patients, and was an independent predictor of SR. Angptl2 deficiency accelerated mesenteric artery thrombosis induced by FeCl_3_ in *Angptl2*^–/–^ compared to WT animals, promoted platelet granule secretion and aggregation induced by thrombin and collogen while purified ANGPTL2 protein supplementation reversed collagen-induced platelet aggregation. Angptl2 deficiency also increased platelet spreading on immobilized fibrinogen and clot contraction. In collagen-stimulated *Angptl2*^*–/–*^ platelets, Src homology region 2 domain–containing phosphatase (Shp)1-Y564 and Shp2-Y580 phosphorylation were attenuated while Src, Syk, and Phospholipase Cγ2 (PLCγ2) phosphorylation increased. Our results demonstrate that ANGPTL2 negatively regulated thrombus formation by activating ITIM which can suppress ITAM signaling pathway. This new knowledge provides a new perspective for designing future antiplatelet aggregation therapies.

## Background

Myocardial infarction is caused by partial or complete blockade of a coronary artery and is a global health challenge with high incidences of morbidity and mortality [1, 2]. ST-segment elevation myocardial infarction (STEMI) is prevalent and is typically caused by aberrant thrombosis leading to vascular obstruction. Primary percutaneous coronary intervention (PPCI) is the standard of care for treating STEMI in order to restore blood flow in the infarct region [3]. Approximately, 15–18% of patients with STEMI undergo favorable spontaneous recanalization (SR) of occluded vessel because of thrombus dissolution prior to PPCI [4–6]. These SR patients recover better than those with persistent occlusion of the arteries [6] and SR is considered to be an independent predictor of favorable outcome in patients with STEMI [5]. Balance between pro-thrombotic and antithrombotic signals play a crucial role in stopping a bleed without causing intravascular thrombosis [5]. Thrombosis is a highly complex process that involves platelets, vessels, and the coagulation system, in which platelets play a key role [7, 8]. Pro-activation and inhibition signals are also present in platelets [8, 9]. Therefore, a better understanding of platelets is of great value for clinical antithrombotic therapy.

Our previous study found that paired immunoglobulin-like receptor B (PIRB), the mouse orthologue of human LILRB2, which contains immunoreceptor tyrosine-based inhibition motifs (ITIMs), inhibits platelet activation pathway initiated by the immunoreceptor tyrosine-based activation motif (ITAM) via the activation of Src homology region 2 domain-containing phosphatases 1/2 (SHP1/2) [9]. As the ligand of PIRB, angiopoietin-like protein (ANGPTL)2, is expressed and stored in platelet α-granules [9]**.** ANGPTL2 was first discovered and cloned in 1999. It is one of seven members of the ANGPTL protein family. Functionally, ANGPTL2 possesses proinflammatory properties and plays a role in the development of a number of chronic diseases, including cancer, diabetes, and atherosclerosis [10–12]. In vitro, purified ANGPTL2 protein inhibited platelet aggregation stimulated by various agonists, such as collagen, thrombin, and adenosine diphosphate (ADP) through its receptor PIRB [9]. However, it remains unclear whether and how ANGPTL2 influences platelet aggregation and thrombosis in vivo.

The objective of this study was to explore the expression of ANGPTL2 in STEMI patients with SR and the role of ANGPTL2 in arterial thrombus formation, and to clarify the possible mechanisms. This will provide new target for the prevention and therapy of thrombosis.

## Materials and methods

### Study design

Patients who developed STEMI and underwent PPCI between August 2016 and November 2019 at the Shanghai Ninth People’s Hospital, affiliated to the Shanghai Jiao Tong University School of Medicine, were recruited in this study. Patients were excluded if they had a history of coronary heart disease, late presentation (> 12 h), infectious or inflammatory disease, severe liver or renal disease or thrombolytic therapy. The STEMI diagnosis was confirmed by coronary angiography, which was followed by the administration of 300 mg aspirin, 180 mg ticagrelor, and 0.15 g·kg^-1^·min^-1^ tirofiban infusion. On the basis of the thrombolysis in myocardial infarction (TIMI) flow grade, patients who underwent PPCI were divided into two subgroups: the SR group and non-SR (NSR) group. The observational clinical study complied with the Strengthening the Reporting of Observational Studies in Epidemiology (STROBE) recommendations [13]. All patients provided informed written consent prior to sample collection, and the study protocol was approved by the Shanghai Ninth People’s Hospital Institutional Ethics Committee (HKDL2017300) (No. 2016-256-T191) and performed in accordance with the ethical standards outlined in the 1964 World Medical Association Declaration of Helsinki.

### Plasma preparation and ANGPTL2 quantification

After informed consent was obtained, blood was collected from the patients into plasma sodium citrate anticoagulant tube prior to medication. Plasma fraction was obtained by differential centrifugation at 3000 rpm for 10 min at 26 ℃. ANGPTL2 levels were measured using an enzyme-linked immunosorbent assay kit (E1919Hu, USCN Life Science Inc., Wuhan, China) according to the manufacturer’s instructions. The absorbance at 450 nm was measured using an Infinite M200PRO microplate reader (Tecan, Switzerland, Germany).

### Platelet preparation and measurement of platelet ANGPTL2 levels

Blood was collected from patients into empty syringes moistened with sodium citrate and transferred into polypropylene centrifuge tubes containing 100 μL/mL White’s anticoagulant (2.94% sodium citrate, 136 mM glucose, pH 6.4), 0.1 g/mL PGE1, 1 U/mL apyrase and gently mixed. Platelet-rich plasma (PRP) was fractionated using differential centrifugation (290 g for 10 min). Supernatant was aspirated and the platelets were resuspended with PRP containing 5 mM EDTA and subjected to second differential centrifugation (850 g for 10 min). Washed platelets were resuspended in the modified Tyrode’s buffer as previously described [14].

Platelet ANGPTL2 levels were evaluated immunoblotting as previously described with goat anti-ANGPTL2 antibody (1:1000, AF2084, R&D Systems, USA) and the secondary antibodies (1:3000, #L3042, Signalway Antibody, China) [9]. Image Pro Plus v6.0 (Media Cybernetics, USA) was used to quantify specific protein bands and to determine ANGPTL2 expression levels in platelets. Then data were normalized using the ratio of the band gray value of ANGPTL2 to GAPDH.

### Generation of Angptl2^–/–^ mice

All animal experiments were approved by the Shanghai Ninth People’s Hospital institutional ethics committee (HKDL2017300). Global *Angptl2* deficient (*Angptl2*^*–/–*^) mice were obtained from Jackson Laboratory, while wild-type (WT) C57BL/6 mice genetic background control mice were obtained from Shanghai SLAC Experimental Animal Co., Ltd. *Angptl2* deficiency in platelets was confirmed by comparing western blots of platelet extracts of *Angptl2*^*−*/*−*^ and wild-type (WT) C57BL/6 J mice. All mice were maintained in the Division of Laboratory Animal Resources under specific pathogen free conditions. After the mice were bred, animals homozygous for the null allele were screened and identified. Mice were euthanized with an overdose of isoflurane anesthesia.

### In vivo thrombosis models

Male WT and *Angptl2*^*−*/*−*^mice with a C57BL/6 J background (age, 4–5 weeks old) were used to establish the ferric-chloride (FeCl_3_)-induced mesenteric arteriole injury model. The mice were anesthetized with a ketamine/xylazine mixture (200:10 mg·kg^−1^) via jugular vein cannulation. Rhodamine dye to label platelets was also delivered via this catheter. After the mesentery was exteriorized through a midline abdominal incision, arterioles with diameters of 80–100 μm were treated with filter paper pre-saturated with 250 mM FeCl_3_ for 5 min. The mesenteric arterioles and thrombus formation were visualized using an AxioPlan 2 microscope equipped with an AxioCam MRm camera (Carl Zeiss, Göttingen, Germany). Z-STACK slices of the vessel were captured. Thrombus volumes were determined from the area and height of the clot after the rendered z-stack images were deconvolved with AxioVision Rel 4.6 software (Carl Zeiss, Göttingen, Germany) to produce a three-dimensional image. Thrombus stability was determined by calculating the percentage of the vessel occupied by the thrombus within 2 min and quantified using a score from 1 to 10, with 1 representing 0% to 10% occupancy and 10 representing 91% to 100% occupancy [15, 16]. In these experiments, three mice per group, with 10 thrombi per mouse, were studied.

### Platelet secretion assay

Fresh blood was collected from the abdominal aorta of mice anesthetized via isoflurane inhalation, with syringes containing 100 mL/L sodium citrate anticoagulant (2.94% sodium citrate, 136 mM glucose [pH 6.4]), 0.1 g/mL prostaglandin E1, and 1 U/mL apyrase. Washed platelets were prepared following the above method. The count was read using an automatic blood cell analyzer (BM860, BWLIN MAN, China) and adjusted to 1 × 10^6^ platelets/µL with a modified Tyrode’s buffer.

Platelet secretion was analyzed by measuring the surface expression of α (P-selectin)-and dense-granule, markers of granule release. For α-granule analysis, platelets from WT and *Angptl2*^*−*/*−*^ mice were stained with a fluorescein isothiocyanate-conjugated monoclonal antibody targeting P-selectin (Biolegend, #148305). For dense-granule exocytosis analysis [17], 100 μL of washed platelets (2 × 10^7^/mL) were incubated with 4 μM fluorescent quinacrine (Q3251, Sigma-Aldrich) at 37 ℃ for 10 min. After stimulated by collagen (#385, Chrono-Log), thrombin (T4648, Sigma-Aldrich, USA) or adenosine diphosphate (ADP; A5258, Sigma-Aldrich), platelet secretion was analyzed by flow cytometry using CytoFLEX (Beckman Coulter, USA).

### Platelet aggregation

Then, the washed platelets (300 µL) were used for an aggregation assay in a Lumi-Aggregometer (Chrono-Log, Havertown, PA, USA) [9]. The role played by Angptl2 in agonist-induced platelet aggregation was investigated by stimulating platelets from WT and *Angptl2*^*−*/*−*^ mice with collagen, thrombin or ADP. For further analysis, purified ANGPTL2 was added at concentrations of 0.1, 0.5, and 1 μg/mL to washed WT platelets stimulated with 1 μg/mL collagen, 0.067 U/mL thrombin, or 1 μM/mL ADP. The effect of ANGPTL2 was determined on the basis of decrease in platelet aggregation.

### Platelet spreading on immobilized fibrinogen

Chamber slides with microtiter wells (Nalge Nunc, Rochester, NY, USA) were coated with 50 µg/mL human fibrinogen (Sigma-Aldrich) and incubated at 4 °C overnight. Washed mouse platelets (2 × 10^7^/mL) were stored in Tyrode’s buffer containing 1 mM CaCl_2_ and 1 mM MgCl_2_, and incubated at 37 °C for 90 min. Subsequently, the washed platelets were stimulated with 0.01 U/mL thrombin, and spread on immobilized fibrinogen for 60 min. Then, they were fixed, permeabilized, stained with fluorescein-labeled phalloidin (Invitrogen, Carlsbad, CA, USA), and visualized using an upright fluorescence Axio Scope.A1 microscope (Carl Zeiss, Göttingen, Germany) equipped with a 100 × /1.30 oil objective lens, an X-Cite 120Q light source (EXFO, Mississauga, CA, USA), and a digital camera. At least six images were randomly chosen for each experiment and analyzed under blinded conditions. The platelet surface area was analyzed using Image J software v1.8.0 (National Institutes of Health, Bethesda, MD, USA) [9].

### Platelet-mediated clot retraction

Washed mouse platelets were mixed with citrated normal human platelet-depleted plasma to a concentration of 3 × 10^8^/mL. Subsequently, coagulation was induced using 0.5 U/mL thrombin. The clots were allowed to retract at 37 °C and then photographed at 60 min. Two-dimensional clot size was quantified from photographs by using Image J software v1.8.0, and clot retraction was expressed in terms of the retraction ratio (1–[final clot size/initial clot size]) [9].

### Western blot analysis

Western blot was performed as previously described [9]. Platelets (3 × 10^8^/mL) from WT and *Angptl2*^*–/–*^ mice were stimulated with 1 μg/mL collagen at 37 °C. After the platelets were treated with collagen for 1, 2, 3, 4, or 5 min, they were lysed with radioimmunoprecipitation assay buffer (Thermo Fisher Scientific) on ice for 30 min. Subsequently, they were centrifuged at 13,000×*g* for 10 min at 4 °C to remove cell debris, following which the protein supernatant was stored in a new tube for western blot analysis. The target protein levels were determined using anti-Angptl2 polyclonal antibody (1:1000, #AF 1444, R&D, USA), the secondary antibodies (1:3000, #L3042, Signalway Antibody, China), anti-phospho-Shp1 Y564 (1:1000, #8849, Cell Signaling Technology,USA), anti-phospho-Shp2 Y580 (1:1000, #5431, Cell Signaling Technology, USA), anti-phospho-Src Y416 (1:1s000, #6943, Cell Signaling Technology, USA), anti-phospho Syk Y525 (1:1000, #PA5-104904, Invitrogen, USA), anti-phospho-PLCγ2 Y1217 (1:1000, #3871, Cell Signaling Technology, USA), anti-GAPDH antibody (1:1000, #5174, Cell Signaling Technology, USA) and the secondary antibodies (1:3000, #L3012, Signalway Antibody, China). GAPDH was used as a loading control for total protein.

### Statistical analysis

Statistical analysis was performed using SPSS (v.21.0; IBM Corp., Armonk, NY, USA). Quantitative data were presented as the mean ± standard deviation (SD). Continuous variables were tested for normality by using the Kolmogorov–Smirnov test. The chi-square test was used for categorical variables. Multivariate logistic regression analysis was used to determine the independent predictors of SR. Statistical significance was determined via an independent sample t-test between two groups or ANOVA among multiple groups using SPSS (v.21.0). Statistical significance was set at *P* < 0.05.

## Results

### ANGPTL2 level was correlated with SR in patients with STEMI

**Fig. 1:**
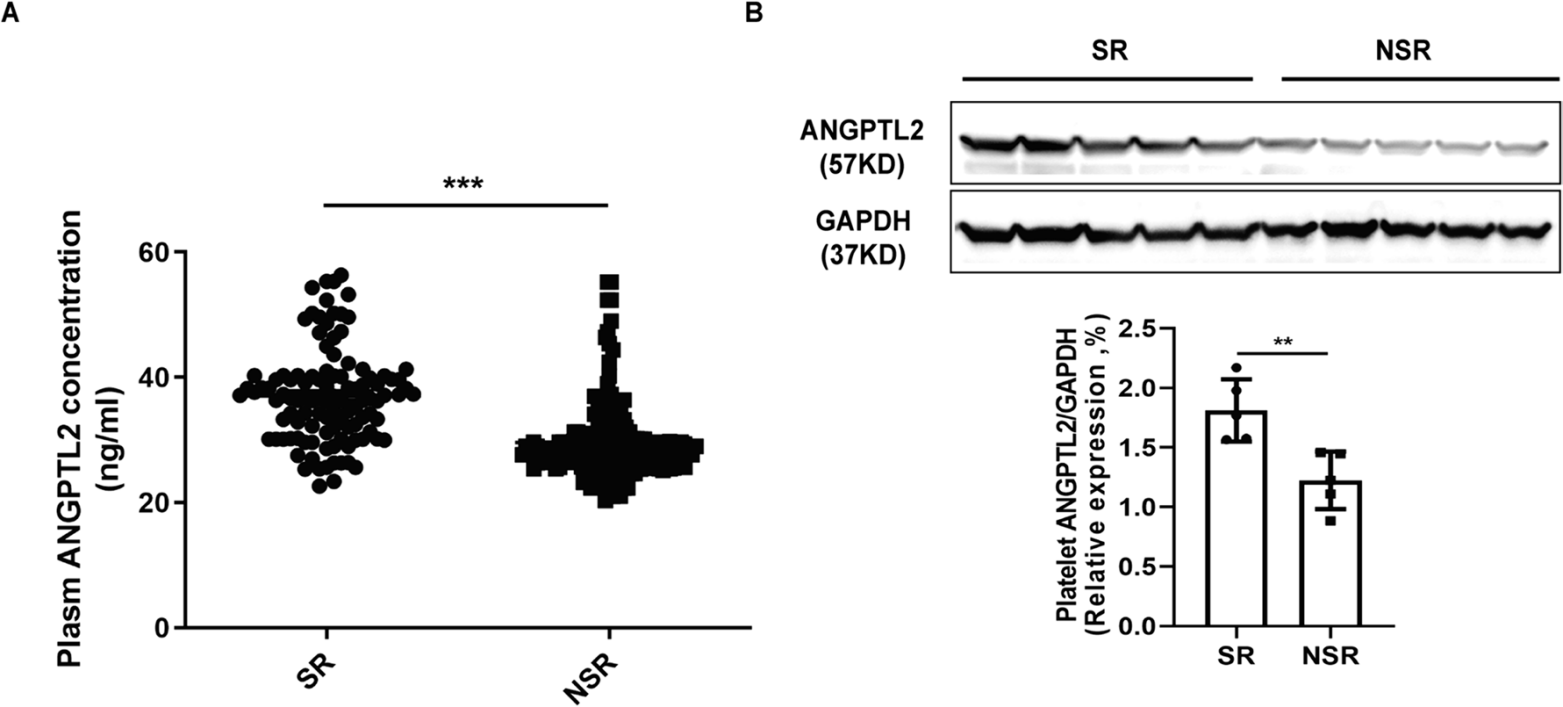
ANGPTL2 expression was higher in patients from SR group than that in NSR group. **A** Plasma ANGPTL2 concentration was higher in patients from SR group than that in NSR group. n(SR) = 108, n(NSR) = 168, ***P < 0.001. **B** ANGPTL2 expression in platelets was higher in SR patients than that in NSR patients. Western blot was performed as described in the Methods. GAPDH expression level served as the loading control. n(SR) = 5; n(NSR) = 5, **P < 0.01

A total of 276 STEMI patients (229 male, 47 female, mean age 64.46 ± 10.66 years old) were included, with 108 SR and 168 NSR patients. The clinical characteristics were shown in Table 1. No significant differences in age, sex and blood pressure were found between SR and NSR groups. However, plasma ANGPTL2 levels in the SR group were significantly higher (*P* < 0.001) than those in the NSR group (Fig. 1A). Multiple logistic regression analysis was therefore performed to determine the independent predictors of SR in patients with STEMI. The plasma ANGPTL2 level (95% confidence interval [CI] 0.828–0.912, *P* < 0.001), uric acid concentration (95% CI 1.005–1.015, *P* < 0.001), and mean platelet volume (MPV) (95% CI 1.773–4.056, *P* < 0.001) were found to be independent predictors of SR Table 2. In resting platelets, ANGPTL2 is expressed and stored in α-granules [9], and when platelets are activated, ANGPTL2 is released and bound to the surface of platelets [9], so platelet ANGPTL2 expression was also measured in patients from both groups using western blot analysis. ANGPTL2 levels were significantly higher in patients with SR (*n* = 5) than those in patients without SR (*n* = 5; *P* < 0.01; Fig. 1B).

**Table 1:** Baseline clinical characteristics findings in patients from SR and NSR groups

Variable	SR (*n* = 108)	NSR (*n* = 168)	*P*
Baseline characteristic			
Age, years	65.92 ± 9.99	63.53 ± 10.10	0.064
Sex, % male	89 (82.41)	140 (83.33)	0.842
Weight, kg	61.62 ± 10.36	60.18 ± 10.30	0.259
SBP, mm Hg	127.51 ± 17.06	124.49 ± 15.35	0.138
DBP, mm Hg	75.58 ± 9.54	76.13 ± 10.85	0.668
Heart rate, beats/min	76.66 ± 9.17	77.92 ± 10.29	0.288
Hypertension, *n* (%)	65 (60.19)	90 (53.57)	0.098
Diabetes, *n* (%)	25 (23.15)	46 (27.38)	0.432
Hyperlipidemia, *n* (%)	47 (43.52)	75 (44.64)	0.854
Smoking, *n* (%)	66 (61.11)	104 (61.90)	0.895
Killip, *n* (%)			0.819
Killip I	93 (86.11)	143 (85.12)	
Killip II	15 (13.89)	25 (14.89)	
Pre-infarct angina, n (%)	44 (40.74)	65 (38.69)	0.734
Laboratory findings			
FBG, mmol/L	6.51 ± 1.50	6.54 ± 1.57	0.880
HbA1C, %	5.81 ± 0.80	5.91 ± 0.76	0.295
Total cholesterol, mmol/L	4.64 ± 0.99	4.67 ± 1.03	0.813
Triglyceride, mmol/L	1.55 ± 0.87	1.55 ± 0.79	0.990
HDL-C, mmol/L	1.12 ± 0.19	1.11 ± 0.25	0.648
LDL-C, mmol/L	2.95 ± 0.79272	2.93 ± 0.84	0.861
Creatinine, µmol/L	78.30 ± 22.31	78.48 ± 23.91	0.949
Uric acid, µmol/L	326.77 ± 62.34	365.36 ± 61.94	0.000
WBC, 10^9^	9.58 ± 2.83	11.34 ± 2.88	0.000
Neutrophils, 10^9^	7.52 ± 2.62796	9.06 ± 2.72	0.000
Lymphocytes, 10^9^	1.41 ± 0.60	1.54 ± 0.66	0.090
Monocytes, 10^9^	0.58 ± 0.22	0.61 ± 0.23	0.289
RBC,10^12^	4.47 ± 0.61	4.52 ± 0.56	0.488
Hemoglobin, g/L	136.21 ± 14.68	139.95 ± 16.68	0.051
Platelet count, 10^9^	201.86 ± 49.88	214.71 ± 54.57	0.065
MPV, fL	10.49 ± 0.75	11.19 ± 0.84	0.000
Coronary artery			
IRA, *n* (%)			0.273
LAD	61 (56.48)	96 (57.14)	
LCX	14 (12.96)	32 (19.05)	
RCA	33 (30.56)	40 (23.81)	
Calcified lesions, *n* (%)	9 (8.33)	21 (12.50)	0.278
Multivessel lesions, *n* (%)			0.517
1-vessel CAD	28 (25.93)	46 (27.38)	
2-vessel CAD	31 (28.70)	38 (22.62)	
3-vessel CAD	49 (45.37)	84 (50.00)	
Plasma ANGPTL2 concentration, ng/mL	37.00 ± 7.56	29.57 ± 6.18	0.000

Hypertension was defined as systolic blood pressure ≥ 140 mmHg and (or) diastolic blood pressure ≥ 90 mmHg; Diabetes was defined as fasting blood glucose ≥ 7.0 mmol/L and (or) 2 hours postprandial blood glucose ≥ 11.1 mmol/L; Hyperlipidemia was defined as total cholesterol > 5.72 mmol/L, or triglyceride > 1.70 mmol/L, or high-density lipoprotein cholesterol < 0.91 mmol/L, or low-density lipoprotein cholesterol > 3.64 mmol/L; Multivessel CAD was defined as the presence of at least 70% stenosis in at least two epicardial coronary arteries

*SR* spontaneous recanalization, *NSR* non-spontaneous recanalization, *SBP* systolic blood pressure, *DBP* diastolic blood pressure, *FBG*, fasting blood glucose, *HbA1C* hemoglobin A1c, *HDL-C* high-density lipoprotein cholesterol, *LDL-C* low-density lipoprotein cholesterol, *WBC* white blood cell, *RBC* Red blood cell, *MPV* mean platelet volume, *IRA* infarct-related artery, *LAD* left anterior descending coronary artery, *LCX* left circumflex coronary artery, *RCA* Right coronary artery, *CAD* Coronary atherosclerotic heart disease, *ANGPTL2* Angiopoietin-like protein 2

**Table 2:** Independent predictors of SR

Variable	B	SE	Wald	P	OR	95% CI
Uric acid level	0.010	0.003	13.277	0.000	1.010	1.005–1.015
WBC count	0.323	0.190	2.894	0.089	1.381	0.952–2.004
Neutrophil count	− 0.170	0.201	0.715	0.398	0.843	0.568–1.252
MPV	0.987	0.211	21.853	0.000	2.682	1.773–4.056
Plasma ANGPTL2 concentration	− 0.140	0.025	32.543	0.000	0.869	0.828–0.912

*SR* spontaneous recanalization, *WBC* white blood cell, *MPV* mean platelet volume, *ANGPTL2* Angiopoietin-like protein 2

### *Angptl2*^–/–^ mice exhibited normal hematopoiesis

To evaluate the role of Angptl2 in vivo, we generated *Angptl2*^*–/–*^ mice. To ensure loss of  *Angptl2* does not affect the hematopoietic system, peripheral blood samples of eight *Angptl2*^*–/–*^ mice and eight sex- and age-matched WT mice were collected and analyzed. As shown in Table 3, we did not observe any alterations in the hematopoietic system including hemoglobin (Hb) level, white blood cell (WBC), red blood cell (RBC), and platelet counts between *Angptl2*^*–/–*^and WT animals (*n* = 8; *P* > 0.05; Table 3).

**Table 3:** Hematologic parameters for wild-type and *Angptl2*^*–/–*^ mice

Hematologic parameter	Wild-type (*n* = 8)	*Angptl2*^*–/–*^ (*n* = 8)	*P*
WBC, × 10^9^/L	7.05 ± 0.82	6.42 ± 0.51	0.088
WBC/Neut/, %	14.23 ± 0.99	14.78 ± 0.83	0.249
WBC/Lymph, %	78.96 ± 3.19	78.53 ± 1.91	0.744
WBC/Mono, %	4.30 ± 0.49	4.49 ± 0.74	0.556
WBC/Neut, × 10^9^/L	1.00 ± 0.11	0.95 ± 0.10	0.339
WBC/Lymph, × 10^9^/L	5.56 ± 0.60	5.04 ± 0.42	0.066
WBC/Mono, × 10^9^/L	0.30 ± 0.05	0.29 ± 0.05	0.578
RBC, × 10^12^/L	8.13 ± 0.36	8.03 ± 0.26	0.517
HGB, g/L	144.88 ± 7.59	138.13 ± 9.46	0.139
PCV, L/L	0.45 ± 0.04	0.41 ± 0.05	0.123
MCV, fL	45.25 ± 3.06	44.75 ± 2.77	0.737
MCH, pg	13.73 ± 0.86	13.28 ± 0.59	0.249
MCHC, g/L	35.13 ± 2.90	33.75 ± 2.92	0.360
PLT, × 10^9^/L	780.75 ± 24.48	776.50 ± 10.65	0.663

Summary of peripheral blood hematologic parameters for wild-type and *Angptl2*^*–/–*^ mice

### *Angptl2*^–/–^ mice displayed enhanced mesenteric artery thrombosis induced by FeCl_3_ in vivo

**Fig. 2:**
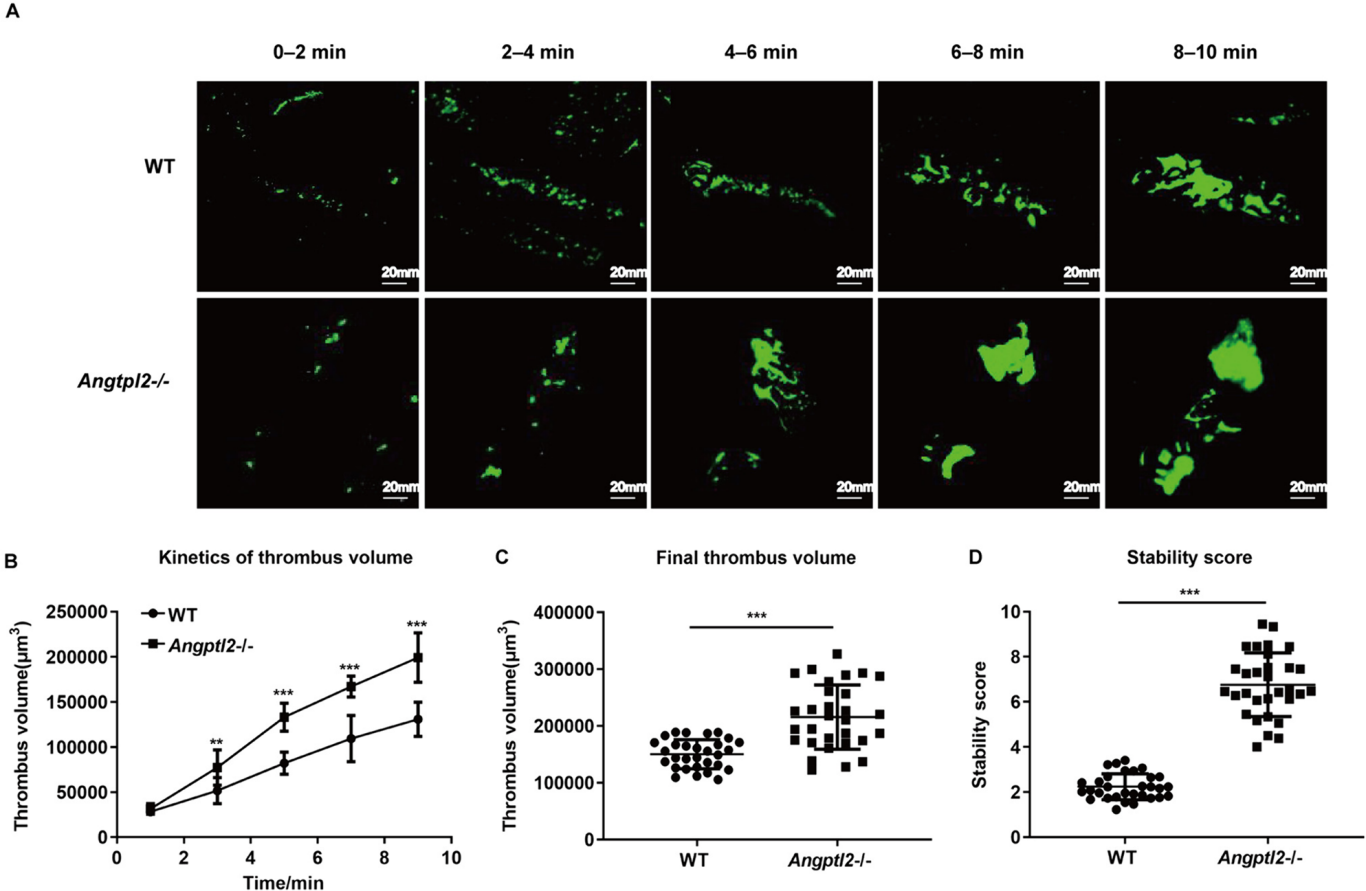
*Angptl2*-knockout (*Angptl2*^*–/–*^) mice displayed enhanced mesenteric artery thrombosis induced by FeCl_3_ in vivo. **A** FeCl_3_-induced mesenteric artery thrombosis was monitored in wild-type (WT) vs *Angptl2*^*–/–*^ mice over 10 min. **B** The thrombus volume in WT vs *Angptl2*^*–/–*^ arterioles over time. **C** Quantitative analysis of thrombus volume at 10 min in WT vs *Angptl2*^*–/–*^ arterioles. **D** The thrombus stability was valued by calculating the percentage of the vessel occupied by the thrombus within 2 min and quantified using a score from 1 to 10, with 1 representing 0% to 10% occupancy and 10 representing 91% to 100% occupancy.  *n *= 29–30 thrombi in each group. * *P* < 0.05, **P < 0.01, ***P < 0.001

To evaluate if Angptl2 participates in thrombus growth, we subjected *Angptl2*^*–/–*^and WT animals to FeCl_3_-induced mesenteric arteriole injury and monitored platelets by fluorescent labeling. Under normal conditions, immediate after vascular endothelium injury platelets rapidly (within 10 min) accumulated as puncta and merge into large thrombus at the injured sites. A total of 29 thrombi selected from 3 WT mice and 30 thrombi selected from 3 *Angptl2*^*–/–*^ mice were evaluated (9–10 per mouse). The thrombus in the injured arterioles of *Angptl2*^*–/–*^ mice developed faster and were substantially larger than those in WT mice (Fig. 2A and B). The volume of the final thrombus was significantly greater in *Angptl2*^*–/–*^ mice compared to WT mice (*Angptl2*^*–/–*^: 21,5529.83 ± 56718.75 μm^3^; WT 149,703.45 ± 25971.98 μm^3^; *P* < 0.001; Fig. 2C). Furthermore, the thrombus was more stable in *Angptl2*^*–/–*^ mice than that in WT mice (stability score: 6.85 ± 1.34 vs 2.27 ± 0.57; *P* < 0.001; Fig. 2D).

### Loss of Angptl2 exacerbated platelet α-and dense-granule secretion

**Fig. 3:**
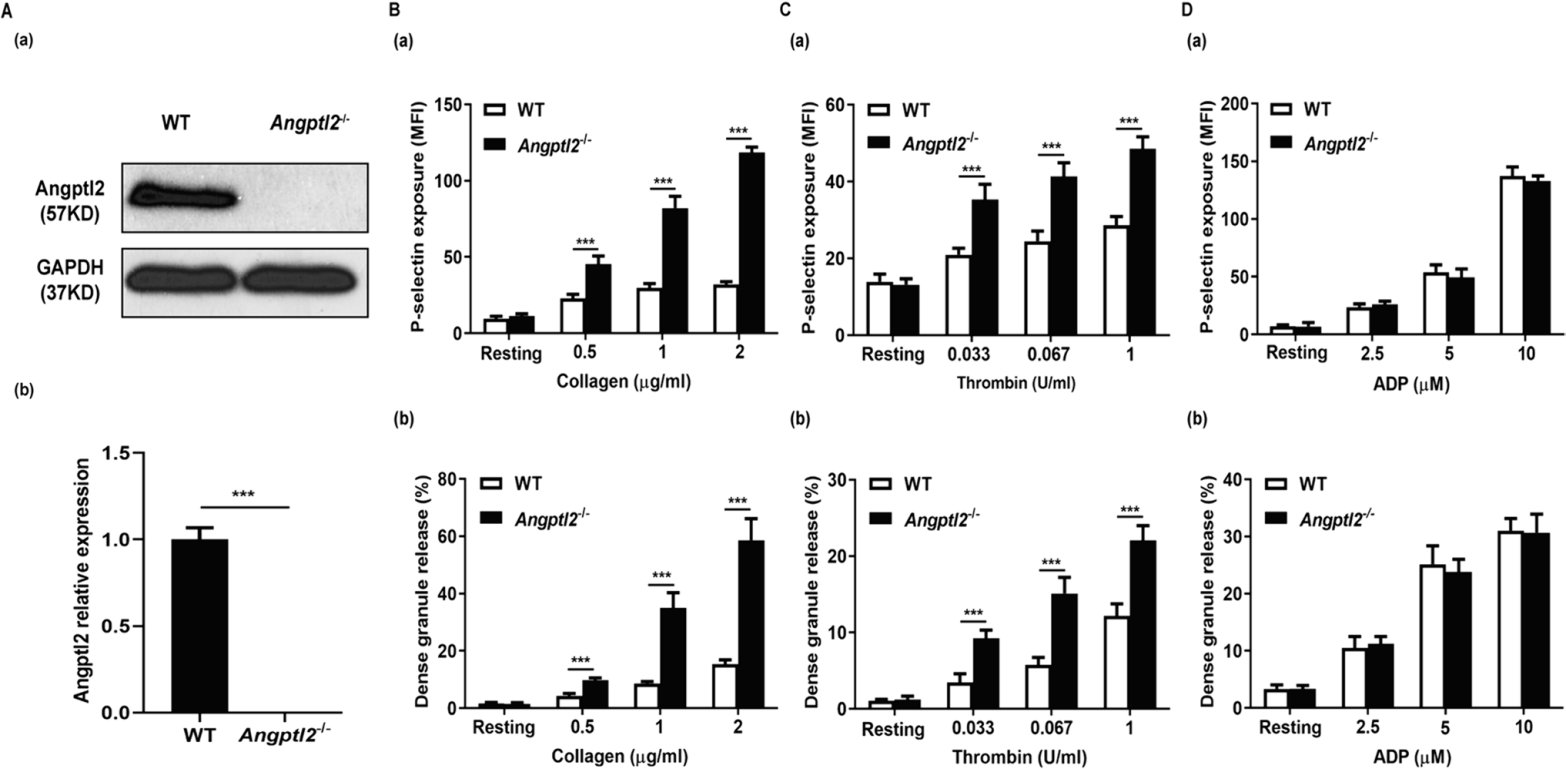
Loss of *Angptl2* exacerbated platelet α- and dense-granule secretion. **A** Western blot results showed that *Angptl2* was depleted in *Angptl2*^*–/–*^ platelets. **B** P-selectin exposure (a) and dense granule release rate (b) measured by flow cytometry after platelet was stimulated with collagen. **C** P-selectin exposure (a) and dense granule release rate (b) after platelet was stimulated with thrombin. **D** P-selectin exposure (a) and dense granule release rate (b) after platelet was stimulated with ADP. *n* = 6. ***P < 0.001

To further clarify the role of Angptl2 in platelet activation and thrombosis, we isolated platelets from *Angptl2*^*–/–*^ mice for further characterization. First, by immunoblotting assay we confirmed the loss of Angptl2 protein expression in *Angptl2*^*–/–*^ platelets (Fig. 3A). Next, we asked if loss of Angplt2 protein affected platelet activation by measuring α (P-selectin)-and dense-granule secretion after collagen, thrombin, or ADP stimulation. Compared to WT platelets, *Angptl2*^*–/–*^ platelets released more α and dense granules when stimulated with collagen and thrombin (*n* = 6; *P* < 0.001) (Fig. 3B, C). However, we observed no difference in α- and dense-granule secretion upon ADP stimulation (Fig. 3D), suggesting Angptl2 mostly responds downstream of thrombin and collagen stimulation.

### Loss of Angptl2 increased platelet aggregation in vitro

**Fig. 4:**
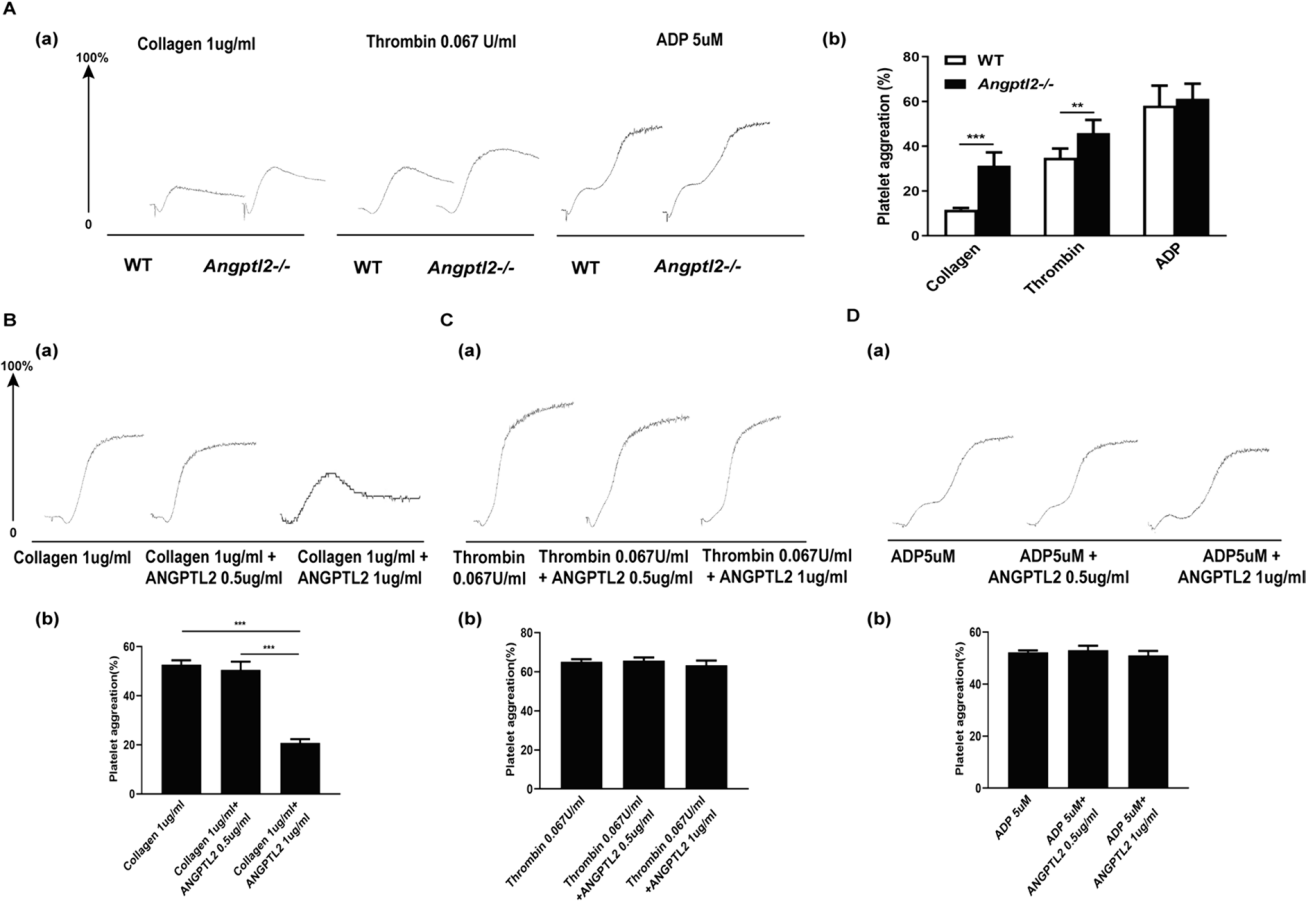
Loss of *Angptl2* increased platelet aggregation in vitro. **A** Aggregation of washed WT and *Angptl2*^*–/–*^ platelets in response to collagen, thrombin, and ADP. (a) Aggregation curve of platelets. (b) Statistical analysis of platelet aggregation rate. **B**–**D** Aggregation of *Angptl2*^*–/–*^ platelets in response to 1 μg/mL collagen (**B)**, 0.067U/ml thrombin (**C)** or 5 μM ADP (**D)**, together with 0.5, or 1 μg/mL ANGPTL2, respectively. *n* = 6. **P < 0.01, ***P < 0.001

Next, we asked if Angplt2 participated in platelet aggregation. To accomplish this, isolated platelets were stimulated with either collagen, thrombin, or ADP and degree of platelet aggregation was evaluated. Angptl2 deficiency resulted in enhanced collagen (*n* = 6; *P* < 0.001)-or thrombin (*n* = 6; *P* < 0.01)-induced platelet aggregation compared to WT (Fig. 4A). Similar to α- and dense-granule secretion results, ADP-stimulated platelet aggregation did not differ between WT and *Angptl2*^*–/–*^ platelets (Fig. 4A). Together, these results indicated that Angptl2 inhibited collagen- and thrombin-induced platelet aggregation.

To further analyze the inhibitory role of Angptl2, purified ANGPTL2 protein was reintroduced to WT platelets at 0.5 or 1 ug/mL concentrations that were stimulated with collagen, thrombin, or ADP and platelet aggregation was evaluated. Addition of ANGPTL2 inhibited collagen-induced platelet aggregation in a dose-dependent manner (*n* = 6; *P* < 0.001) (Fig. 4B). Surprisingly, addition of ANGPTL2 protein did not block thrombin-or ADP-induced platelet aggregation (Fig. 4C, D). The findings suggested that ANGPTL2 inhibited platelet aggregation mainly through the collagen-stimulated signaling pathway.

### Loss of *Angptl2* promoted platelet spreading on immobilized fibrinogen and clot contraction

**Fig. 5:**
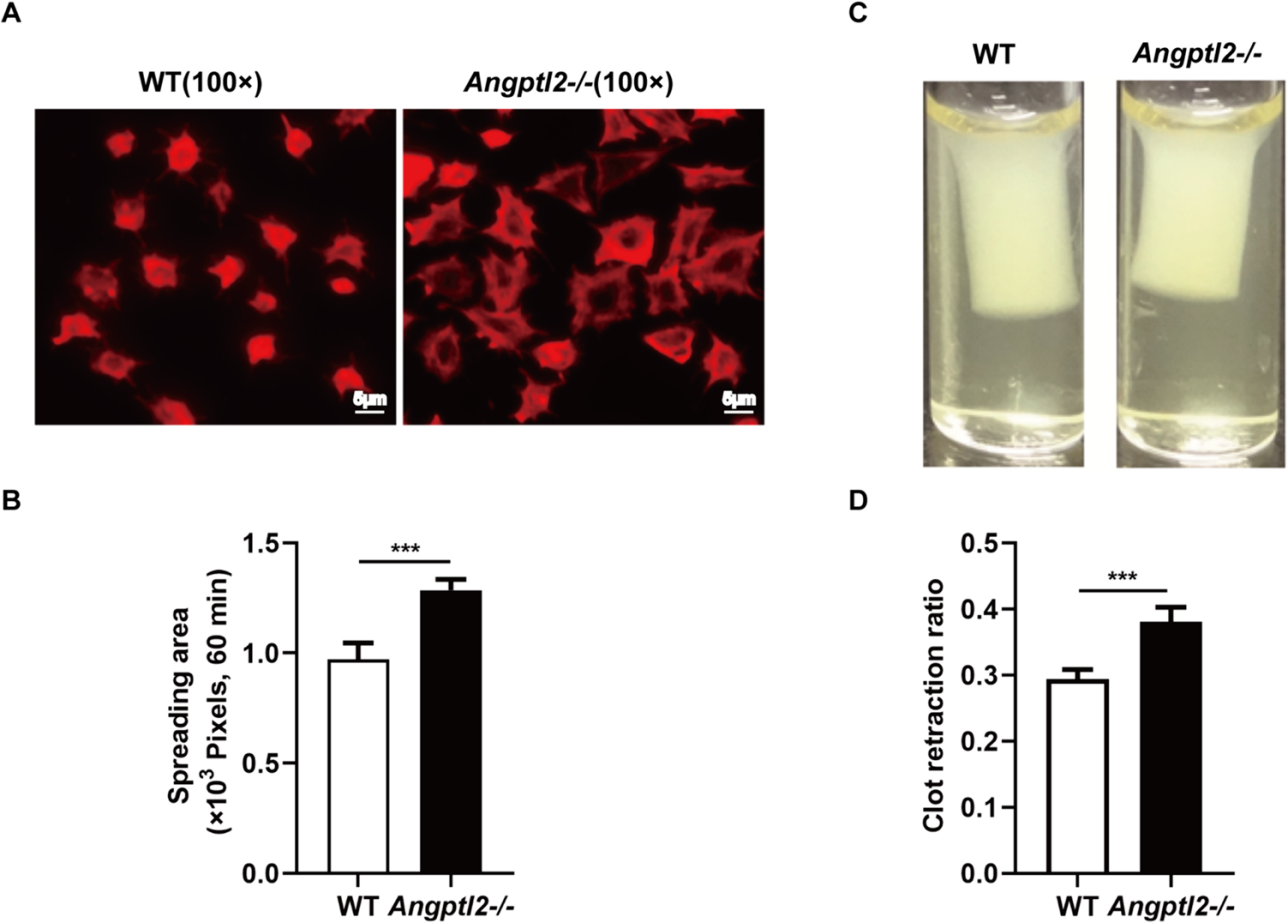
Loss of *Angptl2* promoted platelet spreading on immobilized fibrinogen and clot contraction. **A** Representative phalloidin stained images of WT and *Angptl2*^*–/–*^ platelets spreading on immobilized fibrinogen for 60 min. **B** Quantification of the areas (pixel number) of 6 random fields per experiment. **C** Clot retraction of PRP containing WT and *Angptl2*^*–/–*^ platelets in the presence of 0.5 U/mL thrombin. **D** Two-dimensional retraction of clots was measured, and the data were expressed as retraction ratios. *n* = 6. ***P < 0.001

The binding activity of WT and *Angptl2*^*–/–*^ platelets was evaluated using their static adhesion to immobilized fibrinogen. *Angptl2*^*–/–*^ platelets were found to spread more extensively than WT platelets on immobilized fibrinogen (average area covered in 60 min: WT, 971.17 ± 74.50 pixels vs. *Angptl2*^*–/–*^, 1285.18 ± 49.34 pixels; *n* = 6; *P* < 0.001) (Fig. 5A and B). In addition, Angptl2 is related to clot retraction driven by integrin αIIbβ3. The average clot retraction rate in PRP samples containing WT and *Angptl2*^*–/–*^ platelets were 0.29 ± 0.01 and 0.38 ± 0.02 (*n* = 6; *P* < 0.001), respectively, at 60 min (Fig. 5C and D), suggesting that clot retraction in PRP was accelerated in *Angptl2*^*–/–*^ platelets.

### Angptl2 regulates ITIM and GPVI-ITAM signaling

**Fig. 6:**
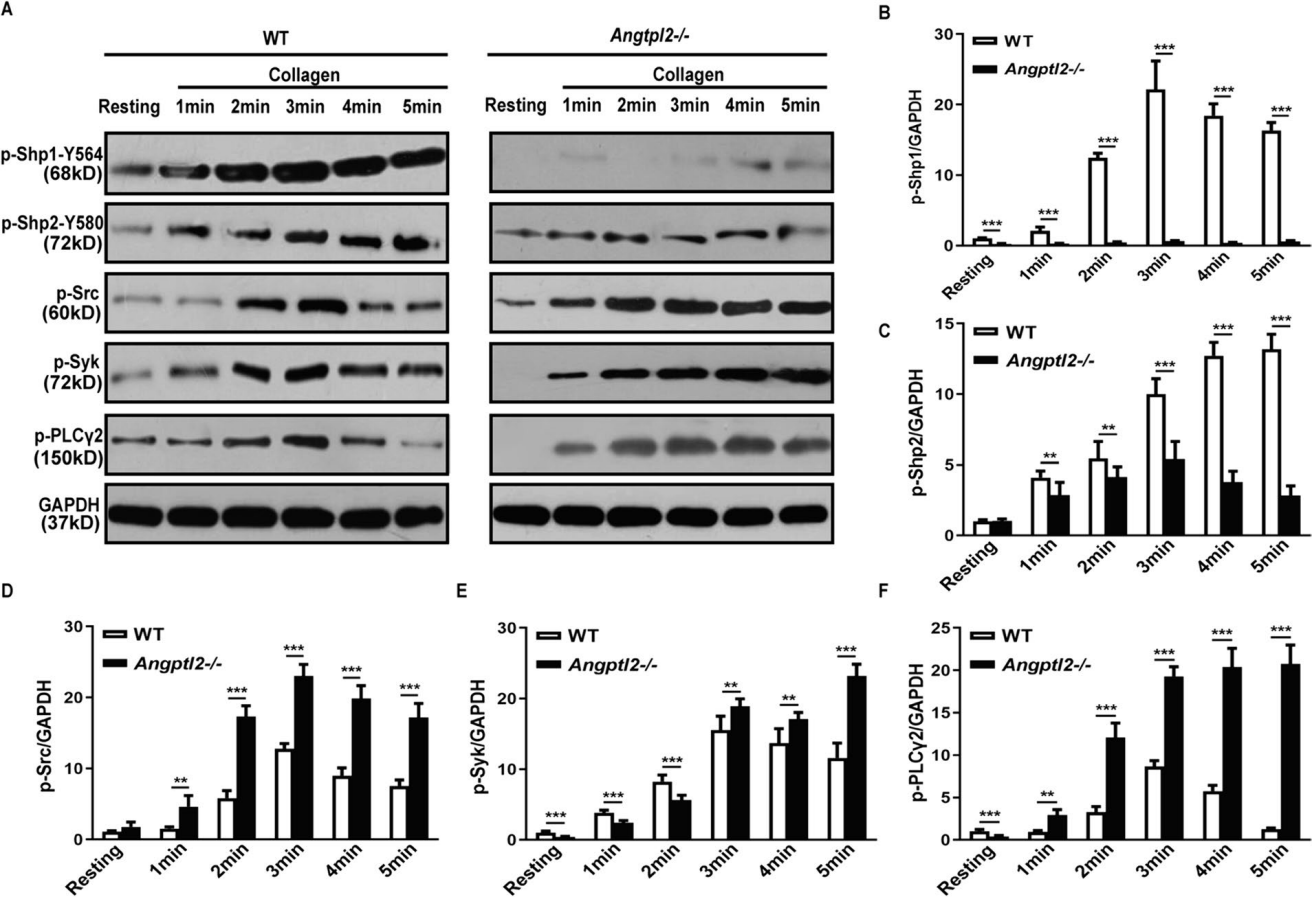
Angptl2 regulated ITIM and GPVI-ITAM signaling. **A** Western blot was performed to measure tyrosine phosphorylation of Shp1, Shp2, Src, Syk and PLCγ2 after WT and *Angptl2*^*–/–*^ platelets were stimulated with 1 μg/mL of collagen. Quantification was conducted to measure the relative level of p-Shp1 Y564 (**B)**, p-Shp2 Y580 (**C)**, p-Src (**D)**, p-Syk (**E)** and p-PLCγ2 (**F)**. *n* = 6. **P < 0.01, ***P < 0.001

To elucidate the mechanisms Angptl2 mediating platelet activity, tyrosine phosphorylation during ITIM and ITAM signaling was measured in WT and *Angptl2*^*–/–*^ mouse platelets stimulated with 10 μg/mL collagen for 5 min. The levels of Shp1-Y564 and Shp2-Y580 phosphorylation in ITIM signaling gradually increased with prolonged stimulation in WT platelets. In contrast, phosphorylation was significantly lower in *Angptl2*^*–/–*^ mice (*P* < 0.01) (Fig. 6A–C).

To determine the role of Angptl2 in ITAM signaling, tyrosine phosphorylation was examined using platelet collagen receptor glycoprotein VI (GPVI)-mediated signaling molecules Src, Syk, and phospholipase Cγ2 (PLCγ2). In WT platelets, the levels of tyrosine phosphorylation of Src, Syk, and PLCγ2 increased from 1 to 3 min, and steadily decreased after that. In *Angptl2*^–/–^ platelets, the levels of tyrosine phosphorylation of Src, Syk, and PLCγ2 were greater than those observed in WT platelets at each detection time point (*P* < 0.01) (Fig. 6D–F). Src, Syk, and PLCγ2 expression levels did not significantly differ in WT and *Angptl2*^*–/–*^ platelets.

## Discussion

The major findings of this study are as follows: (1) plasma and platelet ANGPTL2 levels were elevated in patients with STEMI with SR of the infarction-related artery compared to those in non-SR patients, and were an independent predictor of SR; (2) *Angptl2* knock out accelerated mesenteric artery thrombosis induced by FeCl_3_ in vivo, and the thrombi were more stable in *Angptl2*^*−*/*−*^ mice than those in WT mice; (3) *Angptl2* deficiency promoted platelet granule secretion and aggregation induced by collagen and thrombin, and purified ANGPTL2 protein reversed the collagen-induced; (4) *Angptl2* deficiency promoted platelet spreading on immobilized fibrinogen and clot contraction; and (5) *Angptl2* deficiency inhibited ITIM signaling pathways and activated GPVI-ITAM signaling.

Thrombosis is a highly complicated pathological process in which the vasculature, platelets, and coagulation system interact with each other [7, 8]. Platelet adhesion and aggregation play a key role in this process [7, 8]. Mammalian platelets are mainly derived from bone marrow megakaryocytes. They contain a variety of receptors on their surface, including immunoglobulin receptors, a large group of cell surface proteins that belong to the immunoglobulin superfamily and are involved in cell adhesion, binding, and recognition [18–20]. Many immunoglobulin superfamily members on the platelet surface participate in platelet adhesion, activation, and aggregation [9, 19, 20]. As a typical member of the immunoglobulin superfamily, GPVI has a short cytoplasmic structural domain and lacks a signaling motif [9, 20]. However, it can bind to the ITAM of the Fc receptor γ chain to form a GPVI/FcR γ chain complex, which potently activates αIIbβ3 and causes platelet aggregation, playing an important role in hemostasis and thrombosis [9, 20]. In addition to ITAM-containing receptors, there are many ITIM-containing immunoglobulin receptors on platelet surfaces, such as platelet endothelial cell adhesion molecule-1 (PECAM1) [21] and paired immunoglobulin-like receptor B (PIRB) [9]. With six extracellular Ig structural domains and a cytoplasmic ITIM, PECAM1 weakly inhibits human or mouse platelet activation induced by collagen, ADP, or thrombin [21]. Mouse PIRB, a homolog of human LILRB2, is also an ITIM-containing immunoglobulin receptor [9, 22]. According to our previous findings, PIRB and LILRB2 are expressed in mouse and human platelets, respectively [9]. PIRB contains six extracellular immunoglobulin domains and four cytoplasmic ITIMs, and mutations in *Pirb* upregulate platelet activation in mice by suppressing activation of the ITIM signaling pathway and subsequently decreasing recruitment of Shp1 and Shp2 phosphatases [9].

According to the accepted theory [23–32], upon activation by collagen, platelet GPVI translocates into lipid rafts [23], where Src family kinases (SFKs), including Src, Fyn and Lyn [24], bind to the conserved proline-rich region PxxP in GPVI and become activated by phosphorylation [25–29]. Activated SFKs phosphorylate ITAM in the cytoplasmic region of the GPVI/FcR γ chain, which then recruits and phosphorylates splenic tyrosine kinase (Syk) [25–30]. Activated Syk binds to ITAM tyrosine via a tandem SH2 structural domain. Subsequently, the signal is propagated by the activated Syk that phosphorylates the linker for activation of T-cells (LAT). LAT is a transmembrane scaffolding protein with many tyrosine residues. Phosphorylated LAT binds different kinases, such as PI3K (p85/p110), through the SH2 structural domain of the p85 subunit and junctional molecule grb-2-associated binding protein-1 (Gab1) to form a multiprotein complex that activates PLCγ2 [31, 32]. The subsequent rise in the intracellular calcium concentration augments secretion and increases affinity of integrin αIIbβ3, promoting fibrinogen binding, platelet aggregation, and thrombosis [32]. Then, the secondary agonists released from activated platelets, including ADP, thromboxane A2, and 5-hydroxytryptamine, bind to G protein-coupled receptors to activate surrounding resting platelets, synergistically enhancing the response [26]. In addition to phosphorylating ITAMs, SFKs also activate ITIM-containing receptors by phosphorylating ITIM tyrosine residues, which serve as binding sites for the SH2 structural domain of phosphatases such as tyrosine phosphatase SHP1/SHP2 and inositol phosphatase SHIP1/SHIP2, leading to the inactivation of ITAM signaling pathway components [33]. Furthermore, binding of a protein to an ITIM-containing receptor can also isolate the protein from its site of action: for example, the recruitment of the PECAM-1 receptor for p85 prevents p85 translocation into lipid rafts, which reduces PI3K attachment to Gab1 and LAT in lipid rafts and thereby diminishes activation of the PI3K signaling pathway [26, 32–38]. All of these can lead to the inhibition of platelet activation and thrombosis.

Our previous study found that ANGPTL2, a ligand for the ITIM-containing receptors PIRB and LILRB2, was expressed and stored in platelet α-granules [9]. Moreover, purified ANGPTL2 protein inhibited agonist-induced platelet aggregation and spreading on fibrinogen by binding to PIRB and suppressing collagen receptor GPVI and integrin aIIbb3-mediated signaling [9].

In the present study, we found that plasma and platelet ANGPTL2 levels were elevated in patients with STEMI with SR compared to those without SR. Furthermore, ANGPTL2 level was an independent predictor of SR. Consistent with the clinical data, endogenous ANGPTL2 deficiency enhanced mesenteric artery thrombosis induced by FeCl_3_ and improved thrombus stability in mice. These results suggested that ANGPTL2 levels are inversely related to thrombus stability, and that plasma and platelet ANGPTL2 may serve as a clinical predictor of autolytic recanalization. This finding is significant as it may guide the prognosis of clinical patients with acute myocardial infarction and adjustment of antithrombotic drug applications.

To clarify the mechanisms of endogenous ANGPTL2 effects on platelet activation, *Angptl2*^*−/−*^ mice were used. In agreement with our previously reported in vitro observations of the inhibition of platelet activation by purified ANGPTL2, deficiency of endogenous ANGPTL2 increased collagen- and thrombin-induced platelet activation. Granule secretion and aggregation, as well as platelet spreading on immobilized fibrinogen and clot contraction were all elevated by endogenous ANGPTL2 deficiency. However, evidence from platelet aggregation tests suggested that purified ANGPTL2 protein reversed only the collagen-induced platelet activation, demonstrating that ANGPTL2 has a key inhibitory role in collagen-induced platelet activation. In contrast, the mechanism of the thrombin-induced platelet activation is much more complex, and ANGPTL2 is apparently not the most critical regulator of this process.

To explore the mechanisms of ANGPTL2 effects in more detail, we examined changes in the signaling pathway that involves PIRB/LILRB2, which is the receptor for ANGPTL2. In particular, we investigated PIRB/LILRB2-induced ITIM activation and the resulting ITAM signaling pathway inhibition [9, 26, 39, 40]. Western blot experiments showed that ANGPTL2 deficiency led to decreased phosphorylation of SHP1 and SHP2 in the ITIM signaling pathway, as well as increased p-Src (Y416), p-PLCr2 (Y1217), and p-Syk (Y525) expression, indicating that ANGPTL2 deletion inhibited ITIM that blocks the ITAM signaling pathway. Therefore, increased activation of ITAM signaling pathways resulted in more platelet activation and thrombosis.

## Conclusions

Based on the above evidence, we concluded that by binding to the PIRB/LILRB2 receptor and activating the ITIM signaling pathway which inhibit the ITAM signaling pathway, endogenous ANGPTL2 inhibited platelet activation and thrombosis, and reduced thrombi stability. In conclusion, elevated ANGPTL2 expression in plasma and platelets promoted SR of obstructed coronary arteries in patients with acute myocardial infarction. In addition, ANGPTL2 expression level was an independent predictor of SR. The antithrombotic effect of ANGTPL2 provides a new perspective for clinical antiplatelet aggregation therapy. However, the study has some limitations. First, the clinical trial in this study did not carry out long-term follow-up of the included patients, which could not comprehensively analyze the impact of ANGPTL2 on the long-term prognosis of patients. Second, the in vivo thrombosis assay was performed using *Angptl2* systemic knockout mice rather than platelet-specific knockout mice, which could not avoid the potential effects of a systemic *Angptl2*-knockout. In the future, we plan to design more comprehensive clinical trials and use platelet A2-specific knockout mice to better elucidate the role and mechanism of ANGPTL2 in thrombosis.

## Data Availability

The datasets used and/or analysed during the current study are available from the corresponding author on reasonable request.

## Abbreviations

STEMI: ST-segment elevation myocardial infarction
PPCI: Primary percutaneous coronary intervention
SR: Spontaneous recanalization
PIRB: Paired immunoglobulin-like receptor B
ITIMs: Immunoreceptor tyrosine-based inhibition motifs
ITAM: Immunoreceptor tyrosine-based activation motif
SHP1/2: Src homology region 2 domain-containing phosphatases 1/2
ANGPTL: Angiopoietin-like protein
ADP: Adenosine diphosphate
TIMI: Thrombolysis in myocardial infarction
NSR: Non-SR
STROBE: Strengthening the Reporting of Observational Studies in Epidemiology
PRP: Platelet-rich plasma
WT: Wild-type
FeCl_3_: Ferric-chloride
SD: Standard deviation
Hb: Hemoglobin
WBC: White blood cell
RBC: Red blood cell
GPVI: Glycoprotein VI
PLCγ2: Phospholipase Cγ2
PECAM1: Platelet endothelial cell adhesion molecule-1
PIRB: Paired immunoglobulin-like receptor B
SFKs: Src family kinases
Syk: Splenic tyrosine kinase
LAT: Linker for activation of T-cells
Gab1: Grb-2-associated binding protein-1
